# Diabetic neuropathic cachexia: a case report

**DOI:** 10.1186/1752-1947-8-20

**Published:** 2014-01-15

**Authors:** Deeb D Naccache, William B Nseir, Moshe Z Herskovitz, Mogher H Khamaisi

**Affiliations:** 1 Institute of Endocrinology, Diabetes and Metabolism, Rambam Health Care Campus, 12 Halia Street, Haifa 3109601, Israel; 2 Department of Neurology, Rambam Health Care Campus, 12 Halia Street, Haifa 3109601, Israel; 3 Internal Medicine Department, The Holy Family Hospital, haGalil Street, Nazareth 1610000, Israel; 4 Bruce Rappaport Faculty of Medicine, Technion-Israel Institute of Technology, 12 Efron Street, Haifa 3525433, Israel

## Abstract

**Introduction:**

We report a case of the rare entity of diabetic neuropathic cachexia, in order to remind clinicians that these cases still exist.

**Case presentation:**

A 71-year-old Moslem Arab man with type 2 diabetes along with diabetic neuropathic cachexia complicated by a hyperfunctioning autonomous thyroid nodule, and undiagnosed acromegaly came under our care. We report the unique challenges as to what are the priorities to consider in the course of investigation and treatment. This case emphasizes the fast recovery from this remediable disorder, with antineuropathic medication and exogenous insulin to serve as an anabolic hormone on top of its hypoglycemic effect. Shared pathophysiologic aspects of diabetic neuropathic cachexia, cancerous etiologies and acute phase response are discussed.

**Conclusions:**

Diabetic neuropathic cachexia is an integral differential diagnosis, whenever an intense neuropathic pain dominates patient complaints, accompanied with anorexia, weight loss as well as mood and sleep disturbances. This is an original case report of interest to internists, endocrinologists, diabetologists and pain clinic practitioners. Raising the suspicion of diabetic neuropathic cachexia early and concomitant to weight loss investigation, might curtail suffering and prompt early recovery from a severe illness that has a good prognosis.

## Introduction

Since first reported by Ellenberg [1], the hallmark of diabetic neuropathic cachexia (DNC) consists of varying degrees of symmetrical painful sensory neuropathy, as well as varying degrees of symmetrical motor peripheral neuropathy. There are also autonomic neuropathy complaints and findings such as constipation, diarrhea, gastroparesis, orthostatism and impotence.

Complaints of anorexia, profound weight loss/emaciation of fat and lean mass [2] and emotional instability including sleep disturbances are remarkable.

The outcome of DNC, once treated adequately, is the full-to-considerable recovery of weight, emotional instability, sensorimotor and autonomic neurological manifestations. The treatment of DNC consists of the available antidiabetic treatments (oral medications - metformin and sulfonylureas, or exogenous insulin injections) along with a nourishing diet, with variable use of different antineuropathic medications [1-7].

Altogether, the symptoms and signs of DNC prompt a potential underlying etiology of either carcinomatous, toxic or alcoholic neuropathy, porphyria or chronic relapsing Guillain–Barré syndrome [3]. Weight loss causes should be thoroughly investigated, and adequate therapy should be supplied in a considerable time. Hence, DNC still imparts major challenges with regard to diagnosis and management [4].

The starting event and perpetuating course by which DNC evolves are still assumptive. We present an additional case of this rare entity (30 recorded cases, in 18 reports), since case reports are the sole source of understanding the course and treatment of DNC. Our case is rare in: (a) its complexity of management priorities amongst other comorbid states (uncontrolled diabetes, iatrogenic thyroid toxic nodule, and overlooked pituitary acromegaly); and (b) the iatrogenic impact of specific treatments upon existing morbidities.

## Case presentation

A 71-year-old Moslem Arab man with a 29-year duration of type 2 diabetes mellitus, with diabetic neuropathy and nephropathy, presented to our outpatient clinic, after a three-month period of severe neuropathic paresthetic pain in four extremities and his buttocks (saddle paresthesia). The pain estimate was 10/10 visual analog scale (VAS). Concomitantly, he had lost 20kg of weight, felt anorectic, nervous and sad, had insomnia, tremors, a feeling of general coldness, and was suffering from new constipation and orthostatic complaints. Consequent to orthostatism, his antihypertensive medications were reduced to minimum; ramipril was stopped and atenolol was decreased.

At admission, he complained of dry mouth, fatigue, lethargy and a feeling of suffocation. Recent hemoglobin A1c (9.1%) was achieved on 30 unit/day premixed insulin and metformin 850mg trice daily. Other laboratory findings revealed moderate anemia and chronic mild/moderate renal failure (Table 1).

**Table 1:** Abnormal laboratory results at admission

**Parameter**	**Units**	**Result**	**Normal range**
TSH	miu/ml	<0.01	0.35–4.94
FT4	pmol/L	38.09	9–19
FT3	pmol/L	6.3	2.66–5.7
HbA1c	%	9.1	4.0–6.0
hGH	ng/ml	3.9	0.0–0.8
IGF-1	ng/ml	455	18.6–126.2
25OHD3	ng/ml	17.9	30–100
Hgb	gr/dl	12.2	13.5–17.5
MCV	FL	91	80–98
Creatinine mg/dl	1.62	0.5–1.4	

25OHD3, 25-hydroxyvitamin D3; FT, free thyroxine; HbA1c, glycated hemoglobin; Hgb hemoglobin; hGH, human growth hormone; IGF-1, insulin-like growth factor-1; MCV, mean cell volume, TSH, thyroid-stimulating hormone.

A few weeks prior to his visit, he completed negative thorough endoscopic gastrointestinal testing, as well as whole-body computed tomography (CT) scans searching for neoplastic findings. Except for incidental sporadic lymphangiectasis and an arteriovenous (AV) malformation found in the small bowel and a thyroidal nodule, all test results were within normal limits.

He could not recall any changes in facial features, neither had he noticed an increase in shoe size; he attributed his rounded thick fingers to years of manual carpentry.

On physical examination he was pale and distressed. His blood pressure was 124/63mmHg; pulse, 107 beats per minute; body weight, 86.9kg; height, 171cm; and body mass index (BMI), 29.7kg/m^2^. His facial features were coarse with thickened lips and wide nostrils; he had a palmar fine tremor and a systolic murmur of 2/6. Diminished tendon reflexes were observed in four extremities. There was glove and sock hypoesthesia, and distal weakness of the hands with normal strength of the lower extremities. His current illnesses were arterial hypertension; hearing loss; right carpal tunnel syndrome; obstructive sleep apnea; hyperlipidemia; hiatus hernia with reflux; and benign prostatic hypertrophy.

With negative thorough weight loss investigation, major neoplastic disease and inflammatory bowel disease were ruled out. Absent relevant complaints of malabsorption rendered these diagnoses unlikely.

A thyroid nodule was confirmed by sonography, which was 3.3cm in diameter. His thyroid stimulating hormone (TSH) level of 0.19 was reported one month prior to the repetitive CTs (with iodinated contrast media). It became suppressed thereafter, with elevated thyroid hormone levels (Table 1). The thyroid technetium scan showed asymmetrical goiter and low isotope uptake. Thyroid radioiodine uptake (RAIU) was 1% and 10% after 2 and 24 hours, respectively. Thyroidal fine needle aspirate (FNA) was consistent with normal thyroid follicular findings.

Normal findings were detected in his complete blood count, liver and kidney functions tests, B12, rheumatic diseases profile, human immunodeficiency virus (HIV), testosterone, luteinizing hormone (LH), follicle-stimulating hormone (FSH), prolactin, C-reactive protein and transferrin tests.

We hypothesized that this grave neuropathic suffering underpinned a catabolic and inflammatory mechanism to drive significant emaciation, and to finally result in weight loss, anemia and disrupted acute phase reactants (ferritin and iron). Ferritin was high in contrast to a low correspondent iron level. Ferritin levels observed over time showed a prominent decline before iron stores were replenished (Table 2).

**Table 2:** Iron homeostatic parameters at follow-up

**Parameter**	**(Units)**	**Normal range**	**Admission**	**1m**	**4m**	**6m**	**17m**	**21m**
Ferritin	(ng/ml)	21.8–275	359	74	44	53	ND	150
Iron	(mcg/dl)	50–170	49	40	45	56	30	60
Transferrin	(mg/dl)	200–380	221	233	249	ND	205	242

m, month(s).

Neurological consultation included physical examination, electromyography (EMG) and nerve conduction studies to the lower extremities. Electrophysiological studies revealed a pattern that was consistent with sensorimotor axonal polyneuropathy. Gastric solid emptying scintigraphy revealed minimal isotope passage to the small intestine 3.5 hours after ingestion of a labeled meal.

Biochemical parameters, including growth hormone (GH) and insulin-like growth factor-1 (IGF-1) levels, established acromegaly (Table 1); a head magnetic resonance imaging (MRI) scan revealed a small gadolinium-enhanced pituitary mass of 3 × 5 × 3mm.

The patient’s depressed mood appeared to be mainly attributable to his remarkable neuropathic pains. Hence, we attributed his weight loss to the rare diagnosis of DNC, which accounts for profound weight loss, anorexia, orthostatism, disordered mood and sleep disturbances.

We posted control of the neuropathic suffering as the first priority, and ameliorating hyperthyroxinemia as a secondary requirement; we decided that establishing the cause of acromegaly and its respective treatment to be the lowest of the three priorities.

**Figure 1:**
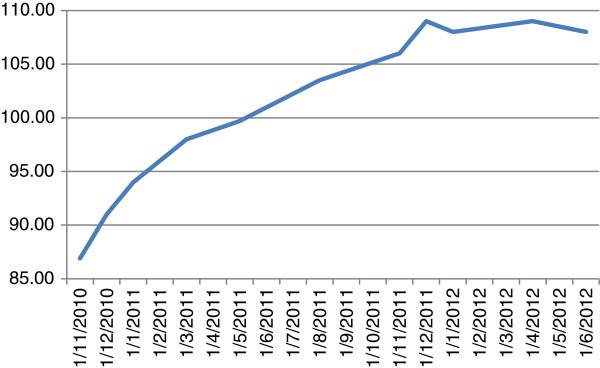
Body weight (measured in kilograms) changes over time.

Weight loss investigation was concluded as negative, after the comprehensive work-up our patient underwent in his local community-based hospital, he denied alcohol intake or past porphyria attacks. Our patient reported a satisfying increase in appetite and gained weight parallel to the decrease in neuropathic suffering (Figure 1). However, infrequently he experienced protracted post-meal vomiting, which did not hamper his weight regain. The gastric emptying test was found to be prominently prolonged as a result of gastroparesis; later on, he experienced an increase in protracted post-meal vomiting once he had been started on a somatostatin analog for acromegaly.

Neuropathy was treated by maximal pregabaline (75mg daily adjusted to serum creatinine) and achieved a pronounced clinical response in respective neuropathic pains. Our patient’s pain estimate dropped to 4 to 5/10 VAS, concomitantly.

Glycemia treatment, including premix insulin doses, was increased up to 70 units in order to keep glycemia relatively under control. This control was achieved parallel to the clinical and laboratory improvement. A hemoglobin A1c of 7.5% three months after admission enabled reconstitution of oral antidiabetics, once glucotoxicity had abated. Metformin was reinstituted and vildagliptin added; both treatments were modified according to the patient’s renal status. Hypoglycemic events prompted a decrease of insulin dosage, and a switch from premix to basal insulin (daily dosage 28 units). Subsequently, our patient experienced a gradual increase in glycemia, which was ascribed to active acromegaly, and the iatrogenic effect of somatostatin analogs on glucose homeostasis (suppressed endogenous insulin).

Hyperthyroxinemia was ascribed to contrast media iodide load, on a preexisting autonomous nodule. His RAIU uptake was respectively low. His thyroid FNA and follow-up function test results were consistent with a benign thyroid nodule. Moderate renal failure was projected in long-term iodide load disposal. However, thyroid function indices dropped spontaneously back to the normal range after eight weeks. His TSH level reverted to pre-evaluation level 16 weeks later.

Acromegaly was hormonally established (Table 1) as a pituitary adenoma-related acromegaly (by MRI). Echocardiography demonstrated negligible mild intraventricular septal hypertrophy.

Our patient preferred a somatostatin analog over transsphenoidal adenomectomy. He was started on lanreotide Autogel® 60mg/month several months after his first visit. Lanreotide failed to achieve good metabolic control: his IGF-1 remained elevated (437ng/ml). Our patient was asked to reconsider pituitary adenomectomy subsequent to the failure of lanreotide, as well as due to the untoward effects of lanreotide (aggravated glycemia deterioration and aggravated gastroparesis-related post-meal vomiting).

## Discussion

The leading presentation was profound weight loss. Notwithstanding, we entertained the intense neuropathic suffering as a leading force in this clinical status. Hence, severe pain management was an appropriate treatment over a reasonable time scale. The intense neuropathic suffering took over from weight loss as the leading presentation; it is unusual to have a complaint eclipsing a substantial physical finding.

Our case brings to mind the importance of physical distress and its consequences. Beyond the scenario of deteriorating diabetes control, physical distress became an etiological factor, rather than a consequence of catabolism-induced weight loss.

Hyperthyroxinemia was detected consequent to imaging tests. Since this rapidly improved, it was considered as a superimposed and innocent thyroid disorder.

Hyperglycemia was relentlessly treated with multiple daily insulin injections. The maximal insulin dose (70 units/day) employed during the peak neuropathic pain was found to be far higher compared to that (28 units/day) needed to achieve an HbA1c level of 7.2% once the neuropathic crisis abated. This decrease reflected a drop in the catabolic impact of pain.

DNC drives general emaciation, implicating the loss of fat and lean tissues [5-7]. Increased ferritin [8] marks an acute phase response similar to that which is seen in surgery stress [9]. Although different in nature, neuropathic pain is very similar to that driven by surgery. The final common pathway observed in surgery includes physical distress and gluconeogenesis from lypolized free fatty acids, as well as protein breakdown [9]; altogether, this comprises the hallmark of emaciation in DNC. Iron is one of the negative acute phase reactants [8]; low serum iron underpins the notion of acute phase response as an executive arm.

Eventually, the decrease in plasma ferritin over time reflected decreasing inflammation in DNC. Ferritin declined to match its corresponding low plasma iron levels (Table 2), rendering itself an unreliable marker of the global iron homeostasis.

Body weight increase reflected a prominent decrease in catabolism, as did a steady decline in glycemia level. A strict diet to control hyperglycemia in circumstances of anorexia is imprudent and inapplicable in a catabolic weight loss state. Catabolism is featured by adipose tissue breakdown with classical symptoms (anorexia and nausea) of its resultant ketonemia [10].

Weight loss here heralded the underlying cause as hyperglycemia, not merely being consequent to glycemic control deterioration due to other reasons.

Lower catabolism means less endogenous glucose production; this is a way of ameliorating pain as a catabolic vector, and intensive insulin treatment as an anabolic vector resulted in weight regain in this case. This course was also reported in a recent similar case report [7].

The acromegaly diagnosis contributed only a little, if at all, to the core management plan, and the specific treatment of acromegaly with a somatostatin analog was started quite late.

Acromegaly as an indolent disease might contribute to the presence of peripheral neuropathy (sensorimotor and autonomic) directly by itself, and indirectly by inducing a consequent diabetic state, which, in turn, can initiate or worsen peripheral neuropathy. The interplay of acromegaly-diabetes-neuropathy is difficult to delineate in terms of which was the primary etiology and which was a major contributor to the consequent neuropathy. Notwithstanding, there are no reports in the medical literature as to an acute course of acromegaly that might lead to catabolic states. On the other hand, our case underlies the need for awareness in routine clinical circumstances (hypertension, sleep apnea, carpal tunnel syndrome), to make a vigilant effort into unifying these conditions in a sole etiology: acromegaly.

## Conclusions

This case report illustrates the need to treat neuropathic suffering early and curb its accompanying catabolic state. Early treatment helped mitigate the urgency to find a neoplastic etiology, and could make investigations of new cases affordable on an outpatient basis. Although it is mandatory to rule out neoplastic etiology, early appropriate treatment is obligatory before establishing etiology.

Our DNC case sheds special light on the order of clinical considerations that govern the complexity of coexisting morbidities: profound weight loss, intense neuropathic pain, uncontrolled diabetes, iatrogenic toxic thyroid nodule and undiagnosed acromegaly, their mutual interaction and priorities of each respective treatment. It also demonstrates the impact of a treatment (lanreotide) of one entity: acromegaly on the control of other coexisting disorders: hyperglycemia of diabetes, and vomiting of gastroparesis.

Our DNC case underpins the good prognosis in treatment of DNC; a prognosis that justifies its early detection and treatment. Finally, DNC is comparable to acute phase response in its catabolic features of emaciation.

## Patient’s perspective

My son did not give up. After making many efforts seeking the diagnosis and advice on treatment, my general practitioner and my internist doctors stated: ‘no way out, we give up’.

My son did not accept that: ‘there must be a way out’. My nephrologist was fair and offered me a referral to consult a diabetologist, a specialist I had never met in my hometown before.

The diabetologist recalled such a case while he was discussing weight loss in a professional meeting with another colleague. The diabetologist did not believe he would see such a rare case before his retirement.

At the end, it paid off remaining stubborn to seek medical advice elsewhere.

## Consent

Written informed consent was obtained from the patient for publication of this manuscript and any accompanying images. A copy of the written consent is available for review by the Editor-in-Chief of this journal.

## Competing interest

The authors declare that they have no competing interests.

## Authors’ contribution

DDN was the consultant diabetologist who wrote the first draft. WN performed the full investigation for gastrointestinal causes of weight loss. MH did the neurological check-up. MK completed the draft and critically revised the case report. All authors read and approved the final manuscript.
